# Understanding the Risk Factors and Stressors Impacting Optimal Work Practices in New Zealand Pharmacies: A S.H.E.L.L Model Analysis

**DOI:** 10.3390/pharmacy11030090

**Published:** 2023-05-23

**Authors:** Lun Shen Wong, Sanyogita (Sanya) Ram, Shane L. Scahill

**Affiliations:** School of Pharmacy, Faculty of Medical and Health Sciences, Grafton Campus, The University of Auckland, Auckland 1010, New Zealand

**Keywords:** community pharmacy, human factors, patient safety, pharmacist, covid model, optimal practice, satisfaction, working conditions

## Abstract

(1) Background: The safe performance of pharmacists is an important issue for patients and regulators. It is recognized that pharmacists interact with a variety of healthcare professionals and act as a bridge between other healthcare providers and systems and patients in the health setting. There has been growing activity in exploring factors that impact optimal performance and determinants that are linked with medication errors and practice incidents. The aviation and military industries have used S.H.E.L.L modeling to identify how personnel interact with factors that affect outcomes. A human factors approach is a useful angle to take when trying to improve optimal practice. Little is known about the experiences of New Zealand pharmacists and S.H.E.L.L factors that affect day-to-day practices in their work environment. (2) Methods: We investigated environment, team, and organizational considerations as the determining factors of optimal work practices using an anonymous online questionnaire. The questionnaire was built from a modified version of the software, hardware, environment, and liveware (S.H.E.L.L) model. This identified components of a work system that were vulnerable and that provided risks to optimal practice. Participants were New Zealand pharmacists approached through a subscriber list provided by the regulatory authority of the profession. (3) Results: We received responses from 260 participants (8.56%). The majority of participants indicated that optimal practice was occurring. More than 95% of respondents agreed that knowledge, fatigue interruptions, complacency, and stress affected optimal practice. Equipment and tools, medication arrangement on the shelf, lighting, physical layout, and communication with staff and patients were important factors for optimal practice. A smaller cohort of participants, 13 percent (*n* = 21), stated that dispensing processes, dissemination, and enforcement of standard operating procedures and procedural guidance did not affect pharmacy practice, 21.3% responded that professional and ethical requirements did not affect optimal practice, 20% stated that having a staffroom affected optimal practice, 20% did not think substance use affected optimal practice, and 30% did not state that cultural differences affected optimal practice. Optimal practice is constrained when there is a lack of experience, professionalism, and communication among staff, patients, and external agencies. COVID-19 also has had an impact on pharmacists both personally and in their work environments. Exploring how the pandemic has affected pharmacists and their work environment warrants further research. (4) Conclusions: Pharmacists across New Zealand agreed that optimal practices were occurring and considered other factors that were perceived to not affect optimal practice. A human factor S.H.E.L.L framework has been used to analyze themes to understand the optimal practice. The rising body of international literature on the effect of the pandemic on pharmacy practice serves as a foundation for many of these themes. Longitudinal data would be useful in exploring some factors, such as pharmacist well-being over time.

## 1. Introduction

The safe performance of pharmacists is an important issue for patients and regulators in New Zealand. It is recognized that pharmacists interact with a variety of healthcare professionals and act as a bridge between other healthcare providers, systems, and patients. Human factor researchers have noted there is growing interest in ascertaining what influences optimal performance and which determinants contribute to medication errors or incidents [1,2]. 

Processes investigating the occurrence of a medication error usually recognize that there are factors outside a pharmacist’s direct control. While pharmacists worldwide have continued to contend with greater workloads, the COVID-19 pandemic has had an impact on pressures in the environment in which pharmacists have previously reported working [3,4,5,6]. Given the abundance of novel and contemporary services provided in pharmacies, such as COVID-19 vaccinations [7,8,9,10], it is pertinent to explore the factors that may affect optimal practice. 

A divergence from optimal performance frequently arises from a series of incidents, with contributions from human and environmental factors. Previous literature in this area is limited and refers to variations in pharmacists or the environments in which they work rather than how these factors interact and what pharmacists perceive as important for the optimal practice to occur. 

A systematic review of the prevalence and nature of medication errors and medication-related harm following discharge from hospital to community pharmacy found that pharmacist interventions alone, such as medicine reconciliation and electronic communication, did not demonstrate a consistent reduction in adverse effects and errors post-discharge [11]. The role and interplay that factors such as staff, equipment, policy, and procedures played in this study are unknown. Another systematic review in a pediatric hospital reports that pharmacist interventions have a generally positive effect on patient outcomes, particularly in areas relating to medication dosing checks [12]. What then makes some pharmacists or environments more likely to reduce risk and errors in their workplaces compared to others? How do we identify what contributes to greater optimal practice given the high-risk nature of the pharmacy work environment?

The aviation and military industries utilize a framework of human factors to investigate incidents, and this may be applied to healthcare to investigate the environment, team, and organizational considerations as potential factors of optimal work practices. The framework looks beyond cause and effect and examines the interaction between factors [13]. Medical practitioners and nurses have utilized this model in risk assessment [1,14,15,16]. A United Kingdom-based researcher proposed that the software, hardware, environment, and liveware (S.H.E.L.L) model can be used to categorize risk determinants to identify components of work procedures that have the capacity to cause risk to optimal practice in pharmacies [1]. 

**Figure 1:**
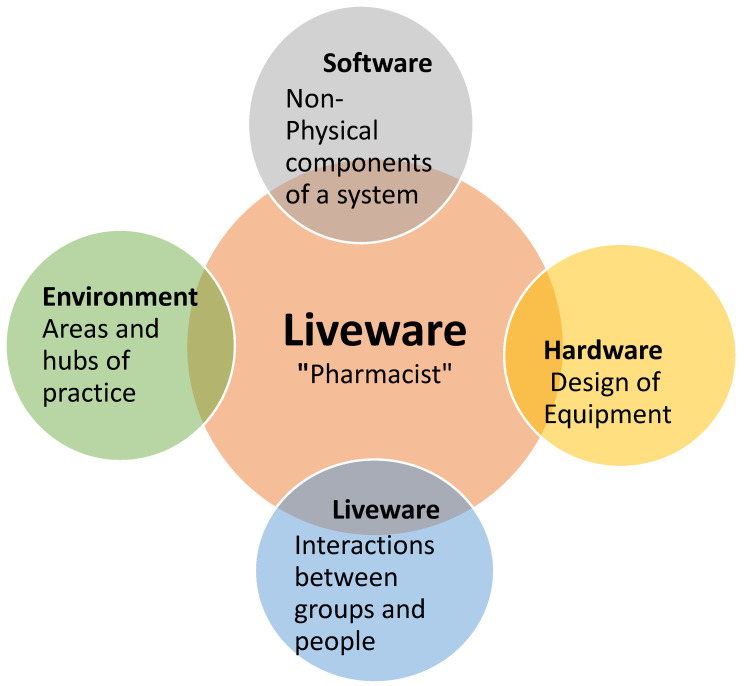
Visual representation of the S.H.E.L.L model.

Unlike other models utilized in assessing risk and errors, the S.H.E.L.L model, as described in Figure 1, provides a more comprehensive understanding of system failures in error management and depicts the inter-relationships present in the current working environment. Software (S) represents interactions with non-physical components of the system, such as checklists, workplace norms, professional standards of practice to be met, and computer systems [1]. Hardware (H) represents the design of the equipment that pharmacists interact with, such as barcode scanners and automated dispensing systems [1]. Environment (E) showcases the areas and hubs of practice pharmacists and includes the tension in community pharmacies with regard to maintaining patient-centered services and business viability [1]. Liveware (L) refers to interactions between two people and accounts for skills in interpersonal communication, interprofessional collaboration, and teamwork within the pharmacy setting [1]. The central piece of this model is represented by another Liveware (L) aspect, which is the most flexible and critical component of the system: the pharmacist who holds a position essential to patient care [1]. The interaction between these factors around the pharmacist as a central factor accounts for the variations in practice that we can see within various pharmacy settings.

The S.H.E.L.L model classifies and helps understand the interplay of human factors in errors that occur in the pharmacy practice setting. It relies on the experience that people make errors, and even in optimal environments, errors can happen and are to be expected [1,15,16]. The model adapted for this paper considered 40 identified risk factors categorized into the S.H.E.L.L model. 

There is limited knowledge of New Zealand pharmacists’ perceptions of optimal practice and what affects it in their everyday working environments. This research examines New Zealand pharmacists’ ideology about the major factors or determinants contributing to optimal factors and where the evidence for this is by utilizing the S.H.E.L.L framework model.

This study aims to investigate the following questions: What do New Zealand pharmacists believe are the major contributing factors to optimal pharmacy practice? What are the S.H.E.L.L factors that affect New Zealand pharmacy practice?

## 2. Materials and Methods

The study was undertaken using a mixed methods approach in a similar manner to the previous publication to answer a query about suboptimal practice in the New Zealand setting [17]. The study gathered data by having registered New Zealand pharmacists complete an anonymous online Qualtrics^TM^ XM survey. Registered pharmacists working in the New Zealand pharmacy sector who have consented to receiving questionnaires from the New Zealand Schools of Pharmacy in relation to their annual practicing certificate renewal were invited to participate by email with a Qualtrics URL survey link. The survey was available from 1 October 2021 until 30 November 2021. No further reminders of the survey were emailed, and no follow-up non-responder bias was examined [17].

### 2.1. Questionnaire Design

The questionnaire was adapted from an aviation industry human factor framework utilized to investigate the potential environment, team, and organizational factors or determinants of optimal work practices [1]. The adapted model created questions categorizing the risk determinants or factors to identify areas of work systems and practices that could potentially pose risks to optimal practice.

The questionnaire comprised three sections. The demographic data and the perceived factors influencing optimal practice were gathered in the first two sections, while the third investigated the pharmacist’s working environments and their perspective of what was important to them in their workplace practices. A Likert scale to investigate determinants of potential risk and the degree to which they concurred or contradicted with what occurred in their workplace assisted this inquiry. The third section investigated the interactions between software, hardware, environment, and liveware (S.H.E.L.L) [1]. 

Eight pharmacists were invited to participate in the survey pilot. Modifications to the gender identity queries to include “non-binary” and “prefer to self-describe” options were added. Wider ethnicity options and workplace title demographics to accurately represent current practicing environments were altered. To address further concerns about the framework and its ability to be applied to different workplace settings resulted in additional modifications being made.

The analytical software, the IBM Statistical Package for the Social Sciences (IBM SPSS version 19), was utilized for the quantitative data analysis. Frequency data according to participant characteristics were utilized for the descriptive statistics [18]. 

Inductive methods were used to analyze qualitatively open-text comments submitted by questionnaire participants before the topics were classified [19]. The thematic analysis outlined the following steps, which were employed to describe familiarity with data, searching for themes to collate into subthemes and generating codes systematically across the data set. During routine research meetings, these were tabulated and reviewed to check consistency across the data set. Two levels of thematic review occurred: using question or topic and using S.H.E.L.L component analysis. Color encoding and circular diagram representations were employed for data presentation. Themes were then defended through scholarly argument, examination, and rationalization in research team meetings [17]. The overarching narrative is presented as a collection of general topics which overview the absolute key drivers of optimal practice. Following this was a visual illustration that is presented in the discussion.

### 2.2. Ethics Approval

The study was approved by the University of Auckland Human Participants Ethics Committee on 2 July 2021 for three years—Reference Number UAHPEC22669.

## 3. Results

The overall response rate of 8.56% was derived from the 260 eligible pharmacists in New Zealand that engaged with the survey. The survey was sent out to 3039 eligible pharmacists in New Zealand.

### 3.1. Participant Demographics

The majority of respondents comprised staff pharmacists in community pharmacies (*n* = 85, 32.7%), community pharmacy owners (*n* = 51, 19.6%), and community pharmacy managers (*n* = 26, 10%). The next most common respondents were hospital pharmacists (*n* = 36, 13.8%). Table 1 illustrates that more females (*n* = 172, 66.2%) responded than males (*n* = 68, 26.2%). Respondents in the 31–35 years and 51–55 years age range (*n* = 35, 13.5%) made up the larger categories. The majority of participants were in practice 30 years or more (*n* = 90, 26.9%), and 12.7% (*n* = 33) were in practice 6-10 years, with 11.9% having practiced 26–30 years and 10 % had practiced for two years or less (*n* = 28).

### 3.2. Quantitative Analysis

Factors that affect optimal pharmacy practice of statistical significance were divided according to the demographic data of the pharmacist participants. Gender differences identified factors relating to inadequate dispensing processes, dissemination, and enforcement of standard operating procedures (SOPs) or procedural guidance/policy (*p* < 0.02), workplace norms (*p* < 0.01), lack of consistency in barcode scanning and not using barcode scanners as intended every time a medication is dispensed (*p* < 0.05), and communication between pharmacists and pharmacy technicians/pharmacy assistants/senior supervisor/manager/other health professionals in the same workplace as being significant (*p* < 0.01).

Factors that were of significance to the age of participants included workplace norms (*p* < 0.02), substance exposure (*p* < 0.02), familiarity with a given task, previous actions (*p* < 0.02), lack of assertiveness of members within the team (*p* < 0.001), lack of teamwork (*p* < 0.01), and cultural differences (*p* < 0.03). Factors of significance to years of practice included factors relating solely to inadequate training or experience (*p* < 0.05). In the work setting, factors such as the arrangement of computers, printers, barcode scanners, and stationery (*p* < 0.007), fatigue, lack of awareness and inattention, distractions, or interruptions (*p* < 0.00001), complacency (*p* < 0.006), stress (*p* < 0.00001), and communication between staff in the same workplace and between consumers of medicines (*p* < 0.001) were considered statistically significant factors. 

Most pharmacists responded that all factors affected pharmacy practice. More than 95% of respondents agreed that knowledge, fatigue, lack of awareness, inattention, distractions or interruptions, complacency, stress, correct equipment and tools, correct communication tools, medication arrangement on the shelf, lighting, physical layout, and communication among pharmacists, staff, and consumers affected optimal practice. 

Almost all respondents (*n* = 159, 99.4%) agreed that fatigue, lack of awareness, inattention, and stress affected optimal pharmacy practice. Table 2 outlines the ten most agreed-upon factors that affected optimal pharmacy practice. Of the top ten, half the factors listed were statistically significant (*p* < 0.05), suggesting that for New Zealand pharmacists, these were the main factors that affected optimal performance. These included fatigue and lack of awareness and inattention, stress, distractions or interruptions, communication among pharmacists and pharmacy technicians/pharmacy assistants/senior supervisor/manager/other health professionals in the same workplace, and complacency. 

A third of respondents did not indicate that cultural differences affected optimal pharmacy practice, while 21 percent (*n* = 35) indicated that professional and ethical requirements did not affect optimal practice. Table 3 outlines the top ten factors that respondents indicated did not affect optimal practice. There were statistically significant responses by gender to inadequate dispensing processes, dissemination, and enforcement of standard operating procedures (SOPs) or procedural guidance/policy, lack of consistency in barcode scanning, and not using barcode scanners as intended every time a medication was dispensed. Age stratification showed statistically significant responses to substance exposure, lack of assertiveness of members within the team, lack of teamwork, and cultural differences. There were no statistically significant differences in responses to these ten factors based on the pharmacist’s place of work and the number of years the respondents had been in practice.

A smaller cohort of participants, 13.5 percent (*n* = 21), thought that dispensing processes, dissemination, and enforcement of standard operating procedures and procedural guidance did not affect pharmacy practice, 21.3% (*n* = 35) responded that professional and ethical requirements did not affect practice, 26.9% (*n* = 36) did not think that having a staffroom for rest affected practice, 20.3% (*n* = 25) did not think substance use affected practice, and 29.5% (*n* = 41) stated that cultural differences did not affect practice.

### 3.3. Summary of Factors That Affect Optimal Pharmacy Practice S.H.E.L.L Model

#### 3.3.1. Liveware–Software: Pharmacist and Systems

Gender differences identified factors relating to inadequate dispensing processes, dissemination, and enforcement of standard operating procedures (SOPs) or procedural guidance/policy (*p* < 0.02) and workplace norms (*p* < 0.006) as significant factors that affected optimal practice. A factor that was of significance to the age of participants included workplace norms (*p* < 0.02). An important factor when considering years of practice is inadequate training and experience (*p* < 0.05). 

**Figure 2:**
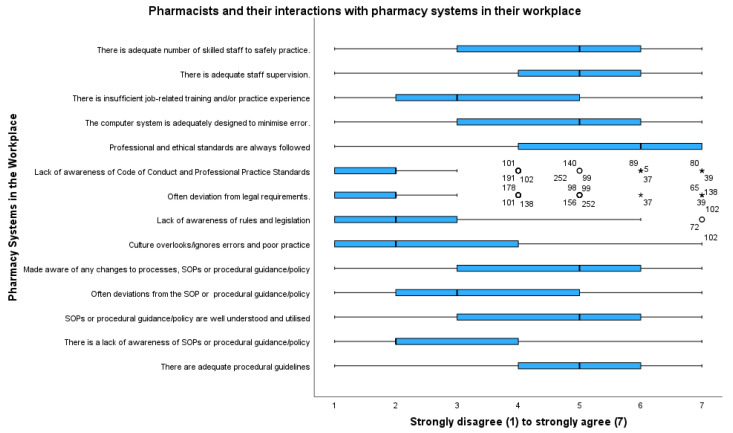
Attitudes towards pharmacists and their interactions with pharmacy systems. Outliers are represented by circles; extreme outliers are represented by asterisks.

Pharmacists’ attitudes and interactions with pharmacy systems, such as workplace norms, software systems, checklists, and legislation, are outlined in Figure 2. Pharmacists strongly disagreed that in their workplaces there was a lack of awareness of the code of conduct and professional standards (m 2.23, mdn 2) and that there was a deviation from legal requirements (m 2.09, mdn 2). However, there was divergence on whether there was a lack of awareness of standard operating procedures or procedural guidance and policy (m 3.11, mdn 3) and whether computer systems are adequately designed to minimize error (m 4.37, mdn 5). As shown in Figure 2, there was divergence on whether there was an adequate number of skilled staff to practice safely, adequate staff supervision, job-related training, awareness of changes to processes, and whether these were well understood.

With regards to software, most pharmacists referenced leadership as a consistent factor that is discussed further in Table 4. 

Minor themes consistent in responses included practice within the law and legislation, a desire for quality improvement, and best practice compliance for audit reviews. Regular error reporting and a hands-on approach from owners and managers were featured in addition to positive sector representation, and innovation within the practice was cited as contributing to optimal practice. Those with 30-plus years of practice (*n* = 41, 26.6%) thought there were inadequate dispensing processes, dissemination, and enforcement of standard operating procedures (SOPs) or procedural guidance/policy. This is possibly due to their vast experiences as practicing pharmacists. 

#### 3.3.2. Liveware–Hardware (L–H): The Pharmacist and Their Physical Workplace 

The arrangement of computers, printers, barcode scanners, and stationery to work settings were noted to be statistically significant (*p* < 0.007).

**Figure 3:**
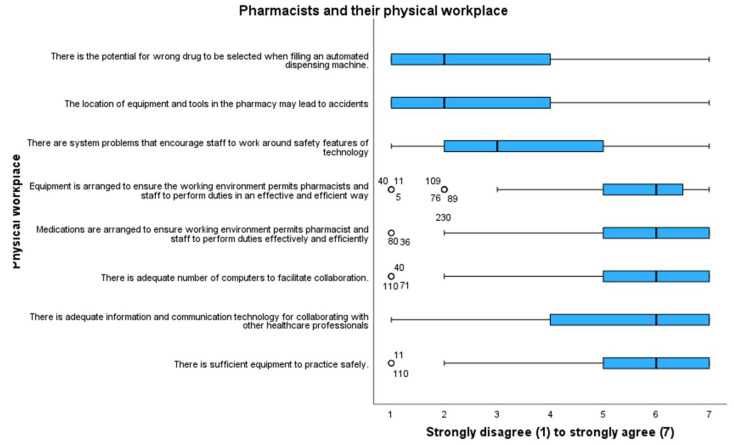
Attitudes of pharmacists and their physical workplace. Outliers are represented by circles.

Figure 3 outlines the pharmacists’ attitudes towards and their interactions with their physical environment and the pharmacy. Pharmacists highlighted that there were system problems in their workplace that inspired staff to work around the safety properties of technology (m 3.59, mdn 4).

Regarding hardware, most pharmacist comments were centralized around the theme of adequate resourcing, represented in Table 4.

Subthemes that progressed from the resourcing theme include adequate pharmacist ratios for working with appropriate support staff that was appropriately remunerated. Training staff and having the right and enough equipment, embracing technology, and having access to electronic databases and adequate stock and inventory for efficient operation were other factors important to practice.

#### 3.3.3. Liveware–Environment (L–E): Pharmacist and the Environment

**Figure 4:**
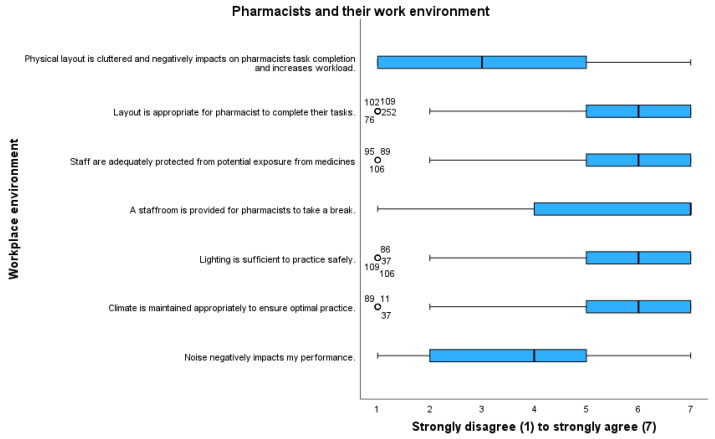
Attitudes of pharmacists and their environment. Outliers are represented by circles.

Age differences identified that substance exposure was a factor that was statistically significant to optimal practice (*p* < 0.022). Gender, years of practice, and work setting did not statistically affect environmental factors significantly. Figure 4 outlines pharmacists’ attitudes toward their interactions with the work environment. 

Most pharmacists agreed that the workplace layout was appropriate (m 5.48, mdn 6) and adequately protected them from potential exposure to medicines (m 5.72, mdn 6). There was divergence in relation to cluttered layout impacting workload (m 3.22, mdn 3) and noise also negatively impacting performance (m 3.55, mdn 4).

With regard to the environment, a consistent topic was the operating environment. This is addressed in greater detail in Table 4.

Subthemes that linked into the operational theme included prioritizing the safety of staff, having robust Standard Operating Procedures (SOPs), and good workflow and timing with adequate teamwork and support. Neatness and tidiness were important to pharmacy design and layout, in addition to adequate lighting and ventilation. A lack of interruptions in task completion, evidence-based practice approaches, and having designated rest periods or breaks were also contributors to optimal practice. Substance exposure to the likes of viruses was identified as a statistically significant factor in optimal practice as a COVID-19 infection was likely to affect a pharmacist’s practice if they were infected.

#### 3.3.4. Liveware (L): Pharmacist

Factors that were of significance to the work setting included factors such as fatigue (*p* < 0.00001), lack of awareness and inattention, distractions, or interruptions (*p* < 0.00006), complacency (*p* < 0.007), and stress (*p* < 0.0001). The age of pharmacists highlighted familiarity with a given task as contributing to optimal practice (*p* < 0.02).

Gender, years of practice, and the majority of age categories did not statistically affect liveware factors significantly.

**Figure 5:**
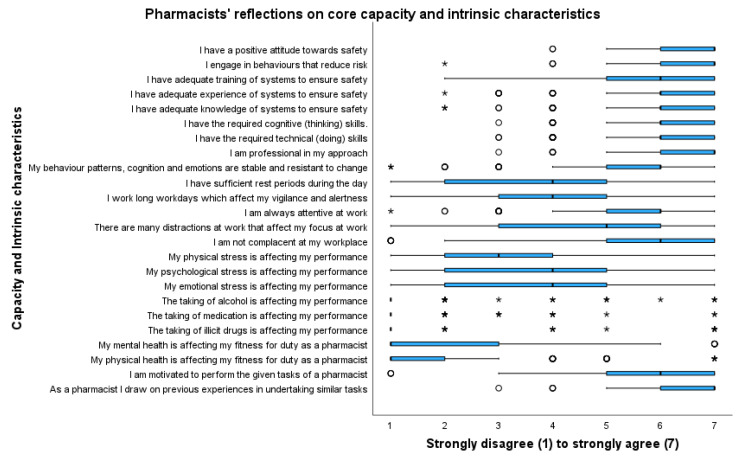
Pharmacists’ reflections on themselves. Outliers are represented by circles; extreme outliers are represented by asterisks.

Figure 5 outlines respondent attitudes to questions related to the pharmacist’s core capacity and intrinsic characteristics. Factors looked at could include knowledge, communication style, personality, cognitive skills, and attitudes. Pharmacists strongly agreed that they had a positive attitude to safety (m 6.53, mdn 7), engaged in behaviors that reduced risk (m 6.37, mdn 7), and had adequate training and systems to ensure safety (m 5.78, mdn 6). However, the pharmacists highlighted that distractions at work might affect their focus (m 4.44, mdn 5), long workdays, which affect their vigilance and alertness (m 4.15, mdn 4), and not having sufficient rest periods during the day (m 3.70, mdn 4) might affect optimal practice.

Pharmacists also highlighted that their physical stress (m 3.12, mdn 3), psychological stress (m 3.69, mdn 4), and emotional stress (m 3.62, mdn 4) affected their performance. 

The importance of personnel was a repetitive topic that was raised in the survey commentary in reference to liveware. Further information is summarized in Table 4. 

Minor themes consistent from looking at personnel included staff that had experience, initiative, passion, high levels of professionalism, and integrity. Personnel who were flexible in their approach, were resilient, and practiced patient-centered care were also linked to optimal practicing environments. In contrast to these optimal factors, pharmacists are working under statistically significant stress (*p* < 0.05) with multiple distractions and interruptions that are not conducive to optimal practice.

#### 3.3.5. Liveware–Liveware (L–L): Pharmacist and Others

**Figure 6:**
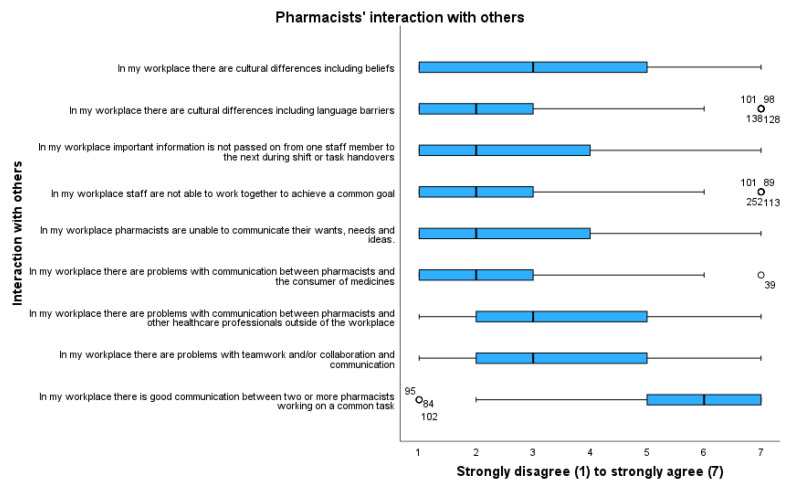
Attitudes of pharmacists towards their interactions with others. Outliers are represented by circles.

Gender differences identified communication between colleagues and other health professionals to be statistically significant (*p* < 0.007). Relationship building is important to any practitioner, and this is supported in the subthemes from Figure 6. Factors that were of significance to the age of participants included assertiveness within teams (*p* < 0.001), the ability of teams to work together (*p* < 0.009), and cultural differences (*p* < 0.028), which highlights the importance of communication as a factor influencing optimal performance. This is reinforced within work settings as communication within established teams and between health consumers are highlighted as important to optimal practice. 

Pharmacists outlined that communication between pharmacists and other healthcare professionals external to the pharmacy could be problematic (m 3.13, mdn 3) and cited challenges with teamwork and/or collaboration and communication (m 3.4, mdn 3).

Communication and relationships continued to be important subjects in relation to liveware–liveware interactions. This is elaborated on in greater detail in Table 4 below.

Subthemes relating to this theme included the maintenance of positive relationships and open communication with district health boards, management, professional bodies, other health care practitioners, and staff. Honesty and trust in communication were also optimal requirements for effective practice. The central role of communication also aligns with the statistical significance of communication between colleagues in the workplace and between health consumers and pharmacists. 

Table 4 below highlights an executive summary of the S.H.E.L.L model themes with associated subthemes and some sample participant quotes to support these. 

## 4. Discussion

This study aimed to understand what New Zealand pharmacists associated with optimal practice and to explore how this applied to their current work settings. Registered pharmacists across all sectors were recruited to explore what they associated with optimal practices in their workplaces and, using a human factor framework, assess which areas of the S.H.E.L.L model affected optimal practice and which did not. To understand pharmacists’ perceptions of the diverse influences encountered that affect their work, a mixed methods questionnaire was used [17]. 

### 4.1. Participant Demographics

Staff pharmacists in community pharmacies (32.7%, *n* = 85) made up the majority of respondents, followed by community pharmacy owners (19.6%, *n* = 51), hospital pharmacists (13.8%, *n* = 36), and community pharmacy managers (10%, *n* = 26. The Pharmacy Council of New Zealand 2021 workforce demographics were comparable to the demographics of the survey respondents [20].

### 4.2. What do New Zealand Pharmacists Believe Are the Major Contributing Factors to Optimal Pharmacy Practice?

Most respondents agreed that knowledge, fatigue, lack of awareness and inattention, distractions or interruptions, complacency, stress, correct equipment and tools, correct communication tools, medication arrangement on the shelves, lighting, physical layout, and communication among pharmacists, staff, and consumers were factors that affected optimal practice.

A minority considered that dispensing processes, dissemination, and enforcement of Standard Operating Procedures and procedural guidance did not affect pharmacy practice, 21.3% responded that professional and ethical requirements did not affect practice, 20% did not think that a staffroom affected practice, 20% did not think substance use affected practice, and 30% did not think that cultural differences affected practice. The role of substance abuse in professional practice was studied in pharmacy student groups, and it is noted that it does occur, which is surprising given the number of respondents who did not perceive this to be an issue [21]. 

There was no statistical difference between years of practice and the responses to these factors in a pairwise comparison; however, there was divergence on whether there was adequate procedural guidance, whether there was an adequate number of skilled staff to practice safely, adequate staff supervision, job-related training, awareness of changes to processes, and whether these are well understood.

The key stressors affecting optimal workplace practice were inadequate staffing, long workdays, distractions, stress, and communication [1,22,23,24]. Pharmacists varied as to whether the computer systems at their workplace were adequately designed to minimize error, raising that there are system concerns that may embolden staff to work around the safety properties of technology.

**Figure 7:**
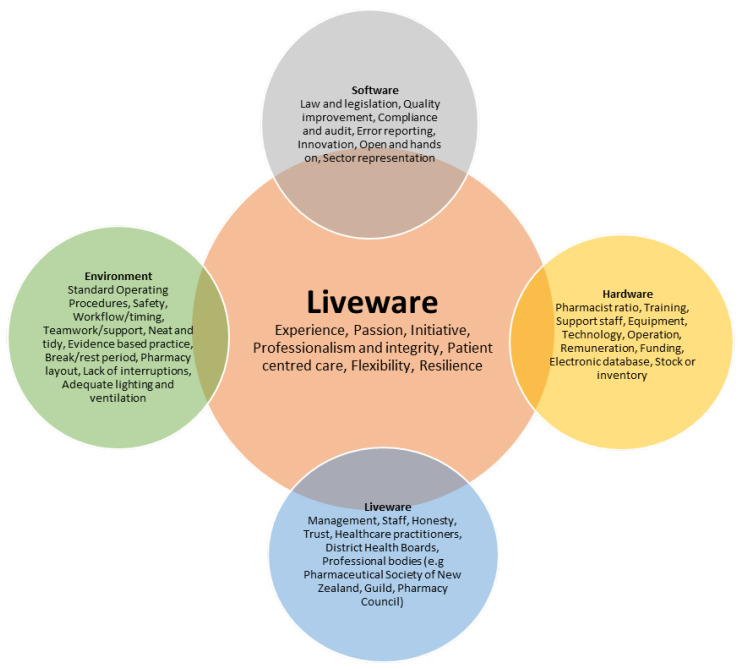
Summarizing what factors pharmacists thought contributed to optimal pharmacy practice.

Figure 7 demonstrates that multiple factors contribute to high-performing humanistic workplaces, and taking a human factor approach allows us to see where multiple vulnerabilities lie [1,2]. A human factor approach also provides opportunities to rank factors and their interactions with moving components, allowing us to implement risk mitigation strategies in a highly targeted manner to increase optimal functioning and research areas where there is a less perceived risk by understanding all the influencing factors [1]. Single factors are not responsible for the optimal practice, and there is a range that provides the landscape and scope for better and safer practices to occur.

Workplace norms and communication within teams were important in contributing to optimal practice from a gender perspective. The literature suggests a higher proportion of younger females in pharmacy, and many of these workers may have younger families and have a dual care burden; both work and home life resulting in fewer full-time work hours [20]. This means that the role of communication between teams and within teams is important as the same team configurations may not be present consistently, so the transition of care between teams is of importance, particularly if you have fewer full-time pharmacists working onsite consistently [25].

In a review of communication between pharmacists and other healthcare professionals, the electronic prescribing systems triggered tensions in communication and highlighted that deviation from compliance standards was a determinant that led to errors and less-than-optimal practice [2]. The factors described as affecting optimal pharmacy practice are also dependent on the pharmacy owners having enough financial means to ensure that in the current market and regulation there is resourcing to offer protected rest periods, reduced interruptions, and allow for stress management and communication within teams and between health users [26,27].

Communication, stress, and rest periods are often strong contributors to errors in practice and the literature [24,27,28]. The data suggests that in stressful periods for the workforce, such as over the pandemic period, these factors are crucial to higher performance.

### 4.3. What Are the S.H.E.L.L. Factors That Affect New Zealand Pharmacy Practice?

There are a variety of factors that contribute to success and optimal practice in pharmacy. The formula for one pharmacy will be different from another due to a range of variables; however, through the analysis, themes emerged across the S.H.E.L.L domains for which local and international literature comparisons can be made. Whilst each pharmacy may have its own challenges in practicing optimally, common factors of interest include work culture, the impact of COVID-19–workload and well-being, leadership, corporatization, and funding and resource. Many of these themes have been reported in previous local studies, and so it is concerning that after more than a dozen years, they continue to surface [3,4,17,28].

The World Health Organization and the International Pharmaceutical Federation (FIP) defined Good Pharmacy Practice (GGP) as a responsive practice to the needs of people who use pharmacists’ provision of optimal and evidence-based care services [29]. Core aspects of this include the ongoing development of relationships and communication with other health professionals and pharmacists. Pharmacy managers and organizations are also required to accept that they have contributed to allowing an environment that was conducive for these practices to occur [29]. Using a human factor framework allows us to holistically identify vulnerabilities in systems; it is a model that goes beyond any currently available definition of good practice because it accounts for a range of factors that each has a role in contributing to practice optimization.

There is no current accepted definition in New Zealand of what optimal pharmacy practice is; however, an analysis based on the S.H.E.L.L framework demonstrates that with respect to Software, good leadership and sector representation, innovations in practice, quality improvement initiatives, and error reporting with compliance and auditing contributes to optimal practice. Workplace norms were an important factor as a proxy measure of whether workplaces completed activities to best practice standards or if there was a culture of deviation from standard practices. Age and years of experience were not statistically important when considered in relation to optimal practice.

Respondents also reported from a Hardware perspective that adequate resourcing should include not only enough and correct equipment but also having enough pharmacists and support staff with continued training and remuneration to contribute to optimal practice. When comparing the arrangement of computers, printers, barcode scanners, and stationery to work settings, this was noted to be statistically significant, too. This would not be out of the ordinary from a work setting point of view as this is likely to affect efficiency, which, in turn, affects optimality.

An environment where there was adequate lighting and ventilation and standard operating procedures that were followed and reflected best practices all contributed to optimal practice. An environment that put safety first, where there was teamwork, support, and break periods, in addition to a lack of interruptions in tasks, also was thought to contribute to optimal practice. From a liveware point of view, communication between management, staff, District Health Boards, and professional bodies were all offered as being important contributors to optimal practice. Experience, passion for the profession, honesty, and initiative were also highlighted as contributing to an optimal practicing environment. The survey data was collected over the COVID-19 pandemic period, where COVID-19 exposure would have an impact on a pharmacy’s ability to practice optimally.

Although our findings are generally positive around optimal practice and what contributes to it in New Zealand pharmacies, there are drivers to suboptimal practice that warrant further research.

### 4.4. Implications of Findings for Practice

The study covers the whole New Zealand pharmacy sector. It is critical to highlight that community pharmacy is currently audited within two frameworks: a full audit with notice against 67 criteria or a standard inspection audit without notice against 10 risk-based criteria. The standard inspection audit, which occurs more frequently, looks at inadequate training and education for medicine supply as an important factor conducive to optimal practice but fails to acknowledge other criteria influencing optimal practice. Some of the optimal factors are subjective to measure; however, the lack of a uniform measurement instrument means that risk-based behavior could be increasing, as reported in Quarter 1 and 2 pharmacy quarter audits from 2021/2022 [30,31]. 

More organizational culture research in pharmacy needs to be undertaken. The literature around culture in pharmacies provides less evidence of the influence of organizational culture on security and caliber in addition to outcomes of service provision. This needs to be quantitated, and effectiveness criteria for pharmacies need to be determined to map culture against them. A human factors approach could be an acceptable tool to capture the dynamic working environment of pharmacists and give more detailed insight into specific interventions that could identify areas to improve community practices [32,33].

Additional research is also required to comprehend the various models of pharmacy and the impact of corporatization on the pharmacy sector in New Zealand. As corporatization has increased in pharmacies, there is little evidence available about the role of corporate structures on safety measures and corporate pharmacies’ efficacy in delivering safe patient-centered outcomes [26]. There is a mass of anecdotal evidence but a scarcity of empirical evidence. Understanding patient and staff outcomes across various models of community pharmacy is warranted to improve our understanding of stress, corporatization, and the role in healthcare from a pharmacy perspective, particularly at a time in New Zealand where access to healthcare providers and discussion around pharmacists’ scope of practices are occurring [34,35].

### 4.5. Limitations

This investigation has several limitations. The survey distribution occurred during the alert Level 4 lockdown in New Zealand, where issues were rife, and the data being collected occurred under challenging conditions. This likely impacted the survey response rate of 8.56%. The sample, in terms of numbers, corresponds with projections of other survey research undertaken in the New Zealand Pharmacy Sector. We recognize there is a likelihood of relatively low generalizability. We understand that there is a risk of ascertainment bias owing to the way in which survey distribution occurred. Pharmacists that declined to provide contact information for research when renewing their annual practicing certificate were not contacted with a survey link. Pharmacists were able to avoid answering survey questions without a return-back function, potentially impacting the accuracy of the response data collected.

In relation to pharmacist well-being, the questionnaire was not cultivated with concerns about pre-existing mental health conditions and their perceptions of how this impacted their day-to-day working time. Investigating matters of prior and current health could have considered benefits when assessing the effect of psychological pressure on pharmacists and if it correlates with optimal practice.

The questionnaire did not discriminate responses received from community pharmacists or owners in reference to whether they were independent, franchise-based, or corporate-owned pharmacies. Additional research concerning the difference in responses between sectors about optimal factors for practice could inform the direction of the business model practice and direct legislative writing and discussion around pharmacy ownership.

## 5. Conclusions

This research aimed to explore the extent to which optimal practice is transpiring across pharmacy practice in New Zealand. The study aimed to understand what factors were associated with optimal practice and whether a human factor approach was a useful tool to help understand factors that affect pharmacy as a high-performance humanistic workplace. 

The S.H.E.L.L model proved to be an effective structure upon which to map the factors that might impact optimal practice. In terms of Software, good leadership and sector representation, innovation in practice, quality improvement initiatives, and error reporting with compliance and auditing contributes to optimal practice. From a Hardware perspective, adequate resourcing, including sufficient and correct equipment, enough pharmacists, and support staff with adequate continued training and appropriate remuneration, contributes to optimal practice. An Environment where there is adequate lighting and ventilation, standard operating procedures that are followed, and reflected best practices all contribute to optimal practice. An environment that puts safety first, where there is teamwork, support, and break periods, in addition to a lack of interruptions in tasks, was also found to contribute to optimal practice. Liveware requirements include communication between management, staff, District health boards, and professional bodies. Experience, passion for the profession, honesty, and initiative were also highlighted as contributing to an optimal practicing environment.

Future research should center on the pharmacy organization and how organizational culture impacts outcomes related to optimal practice. A much more defined research agenda needs to be developed to explore the impact of corporatization globally on the pharmacy sector.

## Figures and Tables

**Table 1:** Participant demographics.

Participant Characteristics	Total
	*n* (*n*%)
Age	
20–25 years	22 (8.5)
26–30 years	31 (11.9)
31–35 years	35 (13.5)
36–40 years	19 (7.3)
41–45 years	16 (6.2)
46–50 years	24 (9.2)
51–55 years	35 (13.5)
56–60 years	29 (11.2)
61–65 years	20 (7.7)
65 and above	9 (3.5)
Total	240 (92.3)
Missing	20 (0.66)
Gender	
Female	172 (66.2)
Male	68 (26.2)
Non-Binary	0 (0)
Prefer not to say	0 (0)
Total	240 (92.3)
Missing	20 (7.7)
Years in Practice	
0–2 years	28 (10.8)
3–5 years	20 (7.7)
6–10 years	33 (12.7)
11–15 years	24 (9.2)
16–20 years	16 (6.2)
21–25 years	17 (6.5)
26–30 years	31 (11.9)
30 and above	70 (26.9)
Total	239 (91.9)
Missing	21 (8.1)
Workplace	Total
Community Pharmacy Owner	51 (19.6)
Community Pharmacy Manager	26 (10)
Community Pharmacist	85 (32.7)
Community Locum Pharmacist	12 (4.6)
Academia	3 (1.2)
Hospital	36 (13.8)
Industry	2 (0.8)
General Practitioner/Primary Health Organization	10 (3.8)
Prescriber Pharm	2 (0.8)
Other	13 (5.0)
Total	240 (92.3)
Missing	20 (7.7)

**Table 2:** Top ten factors that affect optimal pharmacy practice.

Do You Think the Following Factors Affect Pharmacy Practice	Yes (%)	*n*	No (%)	*n*
Fatigue and lack of awareness, and inattention	99.40%	159	0.60%	1
Stress	99.40%	158	0.60%	1
Attitude towards safety	98.70%	157	1.30%	2
Knowledge	97.50%	156	2.50%	4
Distractions or interruptions	97.50%	157	2.50%	4
Lighting	97.30%	146	2.70%	4
Communication between pharmacists and pharmacy technicians/pharmacy assistants/senior supervisors/managers/other health professionals in the same workplace	97.20%	141	2.80%	4
Skills	96.90%	154	3.10%	5
Physical layout of the workplace	96.70%	147	3.30%	5
Complacency	96.30%	154	3.80%	6

**Table 3:** Highest number of respondents stated that these factors did not affect optimal pharmacy practice.

Do You Think the Following Factors Affect Pharmacy Practice	Yes (%)	*n*	No (%)	*n*
Cultural differences	70.50%	98	29.50%	41
Staffroom	73.10%	98	26.90%	36
Professional and ethical requirements	78.70%	129	21.30%	35
Substance exposure	79.70%	98	20.30%	25
Changes to dispensing processes, SOPs, or procedural guidance/policy	80.40%	123	19.60%	30
Lack of assertiveness of members within the team	84.60%	121	15.40%	22
Lack of consistency in barcode scanning and not using barcode scanners as intended every time a medication is dispensed	84.90%	101	15.10%	18
Workplace norms	85.70%	138	14.30%	23
Alcohol, medication, drugs	85.90%	122	14.10%	20
Inadequate dispensing processes, dissemination, and enforcement of standard operating procedures (SOPs) or procedural guidance/policy	86.50%	134	13.50%	21

**Table 4:** Examples of pharmacist quotes in relation to optimal practice.

What Are the Factors Which You Would Associate with OPTIMAL Practice in Your Workplace?			
Framework	Emerging Theme	Sub-Theme	Pharmacist Quote
Software	Leadership and Management	Practice within legislative boundaries Quality improvement Best practice compliance Representation and engagement	“Constant quality improvement of standards…regular identification and mitigation of errors, hands-on involvement from owner… A no-blame culture when errors are made… a focus on the “process” and not the “person” (Pharmacist 4) “Most important is the team culture, if a high standard of work is valued by the team and the leadership then this sets the tone of the workplace.” (Pharmacist 32)
Hardware	Resourcing	Adequate staffing Appropriate remuneration Training and Continuing education Adequate equipment Up to date Information Technology equipment	“Adequate staffing that allows time to complete our work to a high standard, keep abreast of best practice and reflect on how we can improve is vital.” (Pharmacist 32) “Resource management and skill/experience. Community pharmacy is an ad hoc on-call healthcare service provider with income that is derived predominantly from the supply and distribution of medication. An optimal workplace is one that delivers healthcare solutions to customers that meets the illness and wellness needs and expectations of those customers. The ad hoc nature of the service requires flexible resource allocation. That is, human, logistical and product resources need to be coordinated and re-leased in a way that enables effective and timely service provision to our customers. To provide that flexibility one needs a diverse resources and systems (including automation) that enable that release and an economy that enables the provision of those resources.” (Pharmacist 77)
Environment	Operating environment	Staff Safety Standard Operating Procedures Good workflow and design Teamwork and supportive culture Lighting Ventilation Order and tidiness No interruptions	“Having time to accurately dispense, having time to listen to patient’s needs, being able to access doctor’s (both private and public) patient notes to understand what they actually intend, having time to study at work, having time to take a break and sit down during a normal 9 h day.” (Pharmacist 37) “Robust standard operating procedures, good communication within team, physical premises are kept neat, organized and tidy premises is secure and staff are safe from hazards.” (Pharmacist 3). “Collegial support, moderately paced environment, having another pharmacist to double-check and verify prescriptions. Clear communication between colleagues and clear bench space to work with. Organized workspace also helps to optimize workflow and encourages good practice, e.g., placement of folders, caution and advisory labels.” (Pharmacist 47)
Liveware	Personnel	Experience Passion Professionalism and integrity Patient-centered care Flexibility in approach to dealings	“Quality staff who can work effectively at a high level independently, who are confident and competent within their role, who know the role and tasks they do within the team.” (Pharmacist 58) “Friendliness, informative, compassionate service, attention to detail and accuracy and ability to question if concerns arise over prescriptions, ensuring continually updating skills, knowledge and reviewing one’s own practice, enquiring nature and the desire to learn more about the patient, their health and relationships.” (Pharmacist 93)
Liveware-Liveware	Communication and relationships	Honesty Trust External engagement with organizations and professional bodies	“Enough support from colleagues and being able to effectively communicate with doctors regarding clinical issues.” (Pharmacist 43) “Good relationships—to work as a team—within the pharmacy and also with local healthcare providers-e.g., doctors, nurses, physio, hospital.” (Pharmacist 93)

## Data Availability

The data presented in this study are available on request from the corresponding author. The data are not publicly available due to ethical and privacy restrictions.
